# 2-methylbenzoyl berbamine, a multi-targeted inhibitor, suppresses the growth of human osteosarcoma through disabling NF-κB, ERK and AKT signaling networks

**DOI:** 10.18632/aging.103565

**Published:** 2020-07-26

**Authors:** Weixu Li, Yan Li, Wenjia Tian, Xiuguo Han, Jie Zhao, Zengfeng Xin, Hejia Hu, Jun Li, Kai Hang, Rongzhen Xu

**Affiliations:** 1 Department of Orthopedic Surgery, The Second Affiliated Hospital, Zhejiang University School of Medicine, Hangzhou 310009, Zhejiang, China; 2 Orthopedics Research Institute of Zhejiang University, Hangzhou 310009, Zhejiang, China; 3 Department of Endocrinology, The First Affiliated Hospital of Zhejiang Chinese Medical University, Hangzhou 310000, Zhejiang, China; 4 Department of Orthopaedics, Xin Hua Hospital Affiliated to Shanghai Jiao Tong University School of Medicine, Shanghai 200092, China; 5 Department of Orthopedics, Shanghai Ninth People’s Hospital, Shanghai JiaoTong University School of Medicine, Shanghai 200011, China; 6 Cancer Institute, The Second Affiliated Hospital, Zhejiang University School of Medicine, Hangzhou 310009, Zhejiang, China; 7 Department of Hematology, Key Laboratory of Cancer Prevention and Intervention, China National Ministry of Education, Hangzhou 310009, Zhejiang, China

**Keywords:** berbamine, osteosarcoma, apoptosis, migration, proliferation

## Abstract

Osteosarcoma is the most common malignant bone tumor in children and young adults, and it has a survival rate of only 60% with current cytotoxic chemotherapy combined with aggressive surgery. The aim of this study was to evaluate the therapeutic efficacy of the berbamine derivative 2-methylbenzoyl berbamine (BBD24) for osteosarcoma **in vitro** and *in vivo*. We used human osteosarcoma cell lines, primary osteosarcoma cells and mouse models to evaluate the inhibitory effects of BBD24 on osteosarcoma and to determine the molecular mechanism. Our results showed that BBD24 inhibited the growth of the human osteosarcoma cell lines HOS and MG63 in a time- and dose-dependent manner. BBD24 also exhibited significant inhibitory effects on primary osteosarcoma cells. In contrast, BBD24 did not affect normal blood cells under the same conditions. Treatment with BBD24 induced apoptosis, necrosis and autophagy in osteosarcoma cells. Western blot analysis revealed that BBD24 activated the caspase-dependent pathway and downregulated the NF-kB, AKT, and ERK pathways. Finally, BBD24 treatment induced a significant inhibitory effect on the growth of osteosarcoma in nude mice. Our findings indicate that BBD24 is a multitarget inhibitor and may represent a new type of anticancer agent for osteosarcoma treatment.

## INTRODUCTION

Osteosarcoma (OS) is the most common malignant bone tumor that predominantly develops in adolescents and young adults [1]. Even after aggressive chemotherapy and wide excision of tumors, 30–50% of patients with initially localized disease subsequently developed recurrence, which has an extremely poor clinical outcome. Moreover, 20–30% of newly diagnosed patients present with metastatic disease [2, 3]. In addition, patients with metastatic disease and/or relapsed disease show extremely poor survival outcomes [4]. Cisplatin is a standard OS therapy agent that induces OS cell apoptosis; however, changes in the mechanisms that control cell death contribute to resistance to chemotherapy [5, 6]. Recently, increasing evidence has indicated that a number of signaling networks play critical roles in the drug resistance or relapse of OS, including constitutive activation of NF-κB [7–9], ras/ERK [10–12], and PI3K/AKT [13–15] pathways. The metastasis and chemoresistance of OS remain the two main challenges in OS treatment, and it is necessary to improve the curative treatment of OS by identifying novel agents that can disable multiple cancer cell networks and trigger multiple cell death pathways.

Chinese herbal medicines not only play important roles in the discovery and development of drugs but also serve as molecular probes for identifying therapeutic targets. According to a report [16], approximately 70% of all drugs used today for the treatment of cancer are derived from or based on natural products. Arsenic trioxide, homohar-ringtonine and triptolide are three famous examples [17–19]. Berbamine (BBM) is a structurally unique bisbenzylisoquinoline that was isolated from traditional Chinese medicine *Berberis amurensis* and has been used in traditional Chinese medicine for treating a variety of diseases from inflammation to tumors for many years [20–22]. Berbamine and its derivatives have been shown to have potent anti-inflammatory [23, 24] and antitumor activities in diseases including osteosarcoma [25–27]. We previously demonstrated that a new berbamine derivative (2-methylbenzoyl berbamine, BBD24) potently inhibited the growth of imatinib (IM)-resistant chronic myeloid leukemia (CML) cells but not normal hematopoietic cells [28, 29]. However, little is known about the effects of BBD24 on osteosarcoma. To answer this question, the present study used *in vitro* and *in vivo* models to investigate the potential proapoptosis, antimigration and anti-invasion effects of BBD24 in OS cells.

## RESULTS

### BBD24 inhibited the proliferation of osteosarcoma cell lines and primary tumor cells *in vitro*

**Figure 1:**
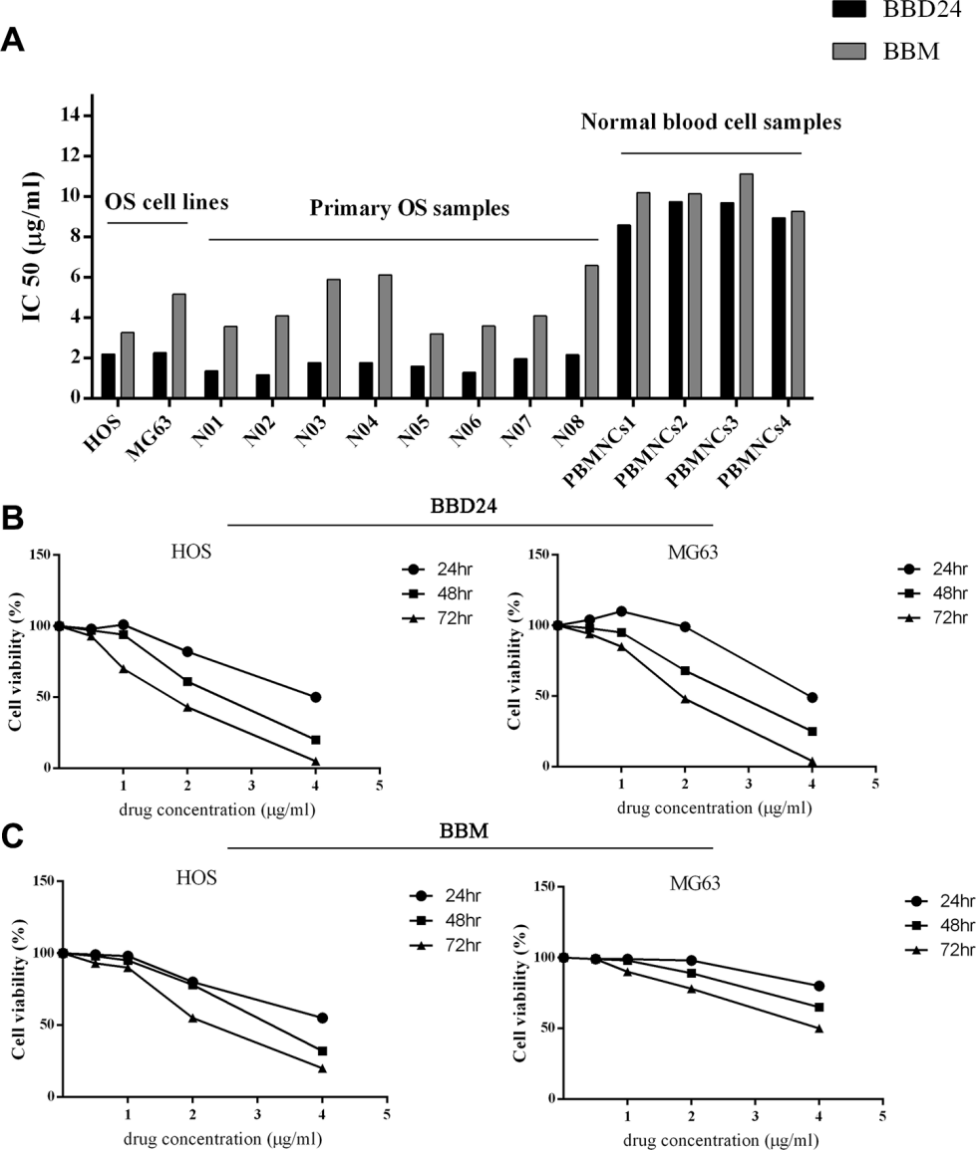
**Effects of BBD24 on proliferation of human osteosarcoma cells and normal blood cells.** Cells were treated with BBD24 at indicated concentrations for indicated times. Cell viability was measured by MTT assay. (**A**) IC50 of BBD24 and BBM on human osteosarcoma cells and normal blood cells (peripheral blood mononuclear cells, MBMNCs). (**B**, **C**) Cell viability of HOS and MG63 after treating with different concentrations of BBD24 and BBM for different times.

We first sought to determine whether BBD24 suppressed the proliferation of human osteosarcoma cell lines *in vitro*. Two human osteosarcoma cell lines, HOS and MG63, were treated with BBD24 at various concentrations. Cell viability was measured using the MTT assay at 24, 48 and 72 hours. The concentration that inhibited 50% of target cells (IC_50_) was calculated from dose-response curves. To investigate the superiority of BBD24 in the fight against OS, we also measured the IC_50_ of BBM in HOS and MG63 cells. As shown in Figure 1, the proliferation of HOS and MG63 cells was greatly inhibited by BBD24 in a time- and dose-dependent manner. The IC_50_ values for HOS cells treated with BBD24 for 24, 48, and 72 hours were 3.88 μg/ml, 2.19 μg/ml and 1.79 μg/ml, respectively (Figure 1A). Similarly, the IC_50_ values for MG63 cells treated with BBD24 for 24, 48, and 72 hours were 3.99 μg/ml, 2.27 μg/ml and 1.90 μg/ml, respectively (Figure 1B). These results suggested that BBD24 had potent antitumor activity against osteosarcoma cell lines, which led us to further investigate whether this compound was also active against primary osteosarcoma cells. Primary osteosarcoma cells were treated with BBD24 at various concentrations for 48 hours. Cell viability was measured by MTT assay, and IC_50_ values were calculated. As expected, BBD24 also significantly suppressed the proliferation of primary tumor cells from all 8 osteosarcoma specimens. The IC_50_ values of BBD24 in the 8 primary tumor specimens ranged from 1.18 μg/ml to 2.17 μg/ml (Figure 1A). In contrast, BBD24 did not affect normal blood cells under the same conditions, and their IC_50_ values ranged from 8.60 μg/ml to 9.75 μg/ml (n=4) (Figure 1A). These results suggested that BBD24 selectively suppressed the proliferation of tumor cells from both osteosarcoma cell lines and fresh primary tumor specimens but spared normal blood cells. The prototype drug BBM was also effective in suppressing OS cell proliferation (Figure 1). However, compared with BBD24, the IC_50_ values of BBM for OS cell lines and primary OS cells were both higher, while the toxicity was similar. Our results indicated that BBD24 was more effective and safer than BBM.

### Effect of BBD24 on NF-κB, ERK and AKT signaling networks

**Figure 2:**
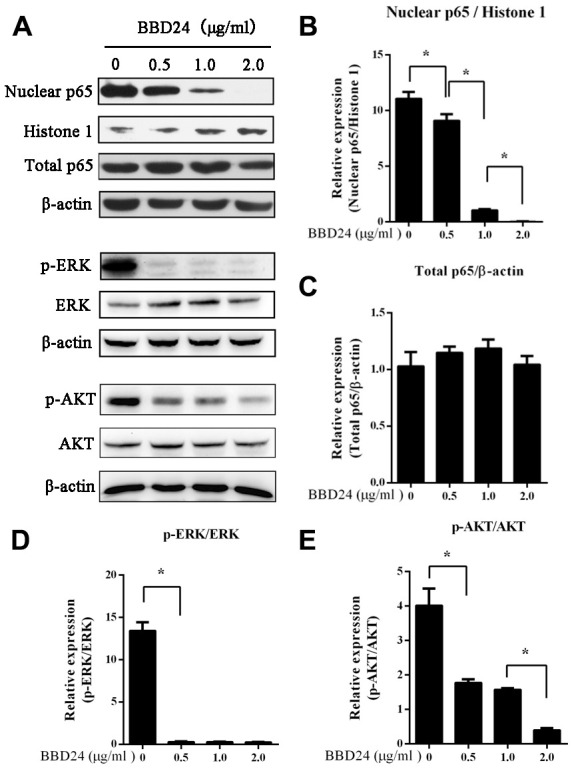
**BBD24 down-regulated NF-κB, ERK and AKT signaling pathways of HOS cells.** The cells were treated with BBD24 at indicated concentrations for 48 hours, and then nuclear and cytoplasmic proteins were extracted for western blot analysis. β-actin and histone 1 were used as cytoplasmic and nuclear loading controls, respectively. (**A**–**E**) BBD24 treatment reduced nuclear NF-κB p65 protein level, and inhibited activation of ERK and AKT of HOS cells in dose-dependent manners. Results were expressed as means ± SD of three independent experiments. * P<0.05.

To further study the mechanism of BBD24 in OS, we investigated whether BBD24 affects the NF-κB and AKT signaling pathways in human OS cells. HOS cells were treated with BBD24 at various concentrations for 48 hours and then collected for analysis of nuclear and total NF-κB p65 protein levels using western blot. We found that treating OS cells with BBD24 caused a significant decrease in nuclear NF-κB p65 protein expression in a dose-dependent manner, but total NF-κBp65 protein expression was not changed substantially (Figure 2A–2C). These results indicated that BBD24 downregulated the NF-κB signaling network by blocking the cytoplasmic-to-nuclear translocation of NF-κB p65 in tumor cells. We next assessed the effect of BBD24 on ERK and AKT activation in human OS cells. HOS cells were treated with BBD24 at various concentrations for 48 hours, and total cellular proteins were extracted for western blotting analysis of total and phosphorylated ERK and AKT. The western blot results showed that BBD24 treatment caused a significant decrease in phosphorylated ERK and AKT protein levels in tumor cells in a dose-dependent manner but did not affect total ERK and AKT protein levels (Figure 2A, 2D, 2E). These results indicated that BBD24 treatment inhibited the activation of both the ERK and AKT signaling pathways.

### BBD24 induced OS cell apoptosis and autophagy

**Figure 3:**
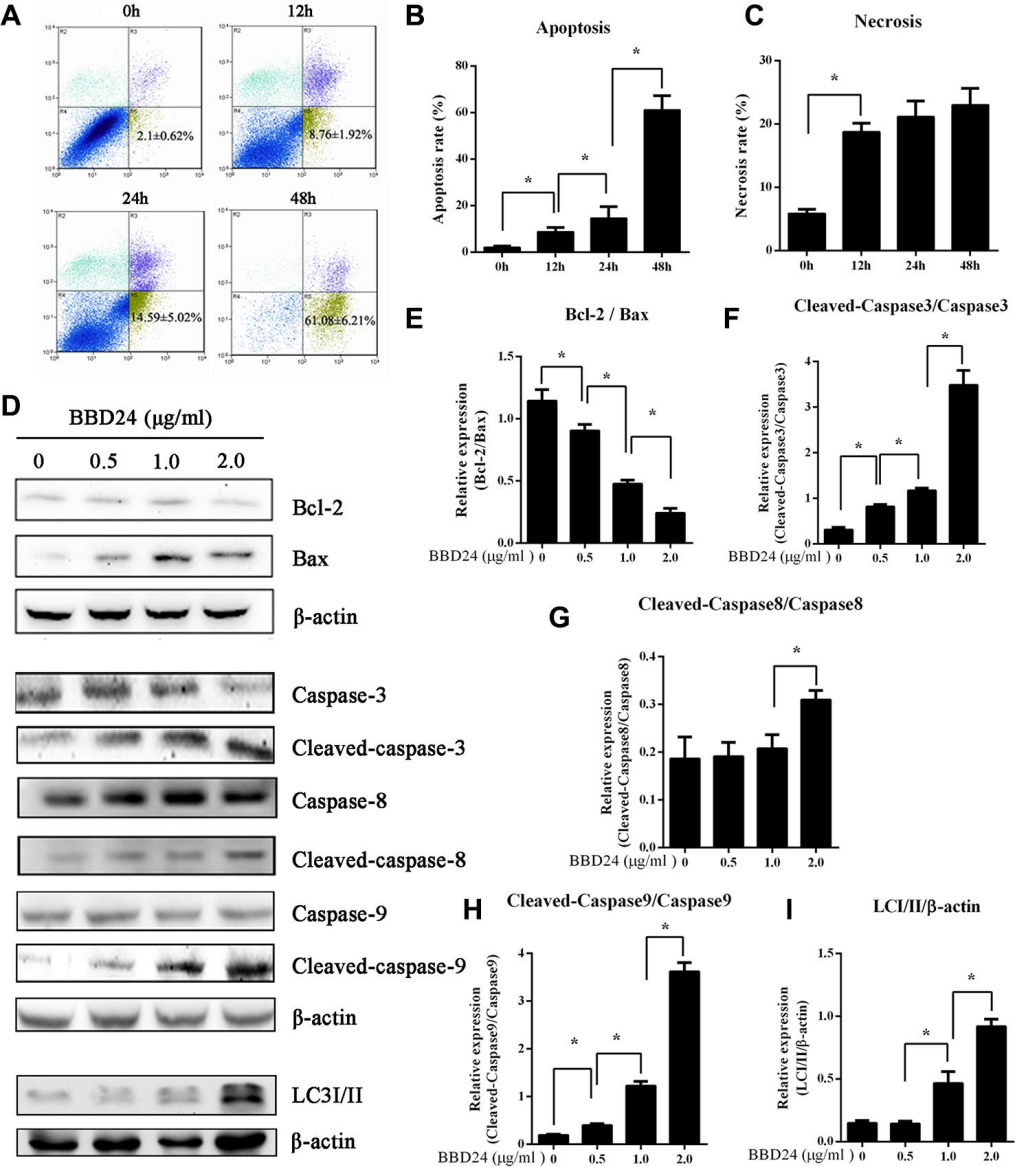
**BBD24 induced multiple cell death pathways of HOS cells.** Human osteosarcoma HOS cells were treated with BBD24 at 2 μg/ml for indicated times. The cells were harvested by trypsinization and collected by centrifugation for analysis of cell viability, apoptosis and necrosis using flow cytometry assay. HOS osteosarcoma cells were treated with BBD24 at the indicated concentrations for 48 hours, followed by Western blot analysis for caspase family and LC3-II. (**A**–**C**) Flow cytometry assay indicated that BBD24 induced apoptosis and necrosis of HOS cells with time dependence. (**D**–**I**) BBD24 promoted activation of caspases and autophagy in HOS cells in a dose-dependent manner. Results were expressed as means ± SD of three independent experiments. * P<0.05.

To establish a link between the above cancer-associated signaling networks and cell death pathways, we further examined OS cell death-related proteins after treatment with BBD24. HOS cells were treated with BBD24 at 2 μg/ml for 0, 12, 24, and 48 hours and then collected for analysis of cell viability, apoptosis and necrosis using flow cytometry (FCM). We found that BBD24 treatment markedly reduced the viability of HOS cells in a time-dependent manner, and we observed a significantly increased apoptosis rate (Figure 3A). Notably, we also found that HOS cells treated with BBD24 for 48 hours displayed greatly increased necrosis (Figure 3A–3C). Western blot analysis provided more information. BBD24 induced Bax overexpression in HOS cells (Figure 3D, 3E). In addition, cleaved caspase-3, cleaved caspase-8 and cleaved caspase-9 were significantly upregulated in BBD24-treated OS cells (Figure 3D, 3F–3H). To determine whether autophagy was also involved in BBD24-induced cell death, we subsequently evaluated the expression level of LC-3I/II in HOS cells after treatment with BBD24 for 48 hours. Interestingly, we also observed a marked increase in LC3 II after BBD24 treatment at ≥ 0.5 μg/ml for 48 h (Figure 3D, 3I). These results indicated that apoptosis pathways were mainly activated along with necrosis and autophagic death pathways in osteosarcoma cells after treatment with BBD24. These findings suggested that BBD24 exerted its antitumor actions by triggering multiple death pathways.

### BBD24 suppressed OS cell migration and invasion

**Figure 4:**
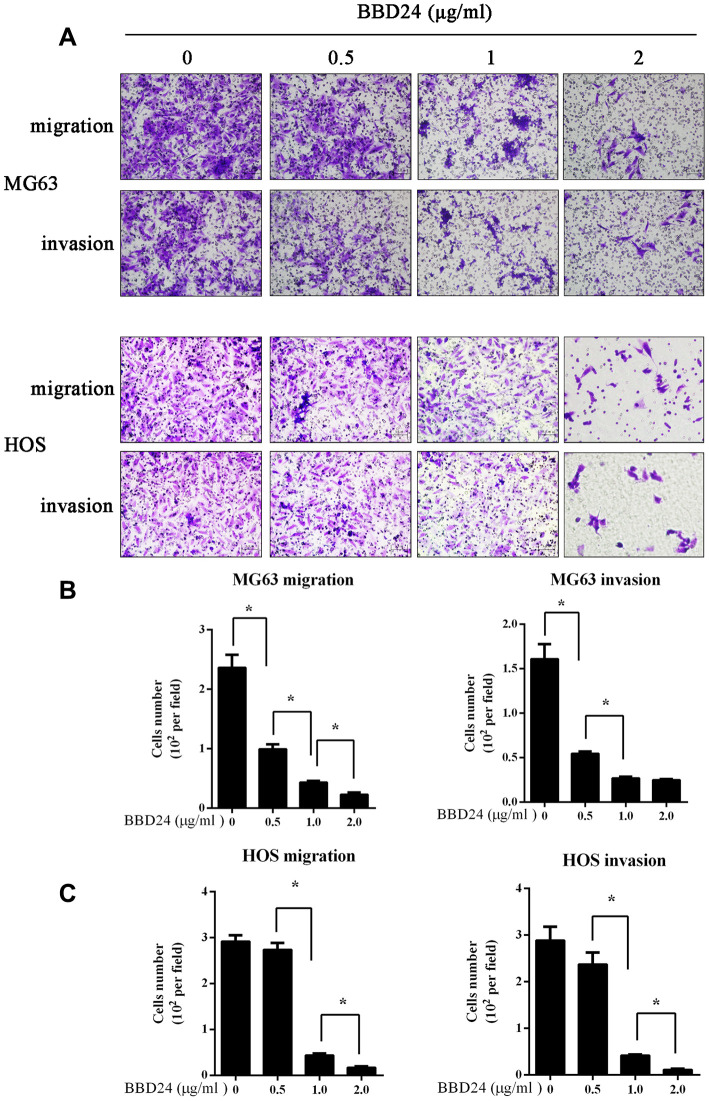
**BBD24 suppressed OS cells migration and invasion.** 1×10^5^ MG63 and HOS cells were seeded on a transwell insert, then treating with different concentrations of BBD24. (**A**–**C**) BBD24 reduced the migration and invasion of MG63 and HOS cells in a dose dependent manner. Results were expressed as means ± SD of three independent experiments. * P<0.05.

MG63 and HOS cells (1×10^5^) were seeded on a Transwell insert to assess migratory and invasive abilities. After treatment with different concentrations of BBD24, the migration and invasion of OS cells were reduced in a dose-dependent manner (Figure 4). Particularly in the 2 μg/ml group, both the migration and invasion of OS cells were almost completely suppressed (Figure 4).

### BBD24 inhibited the growth and increased the cisplatin sensitivity of OS cells *in vitro* and *in vivo*

**Figure 5:**
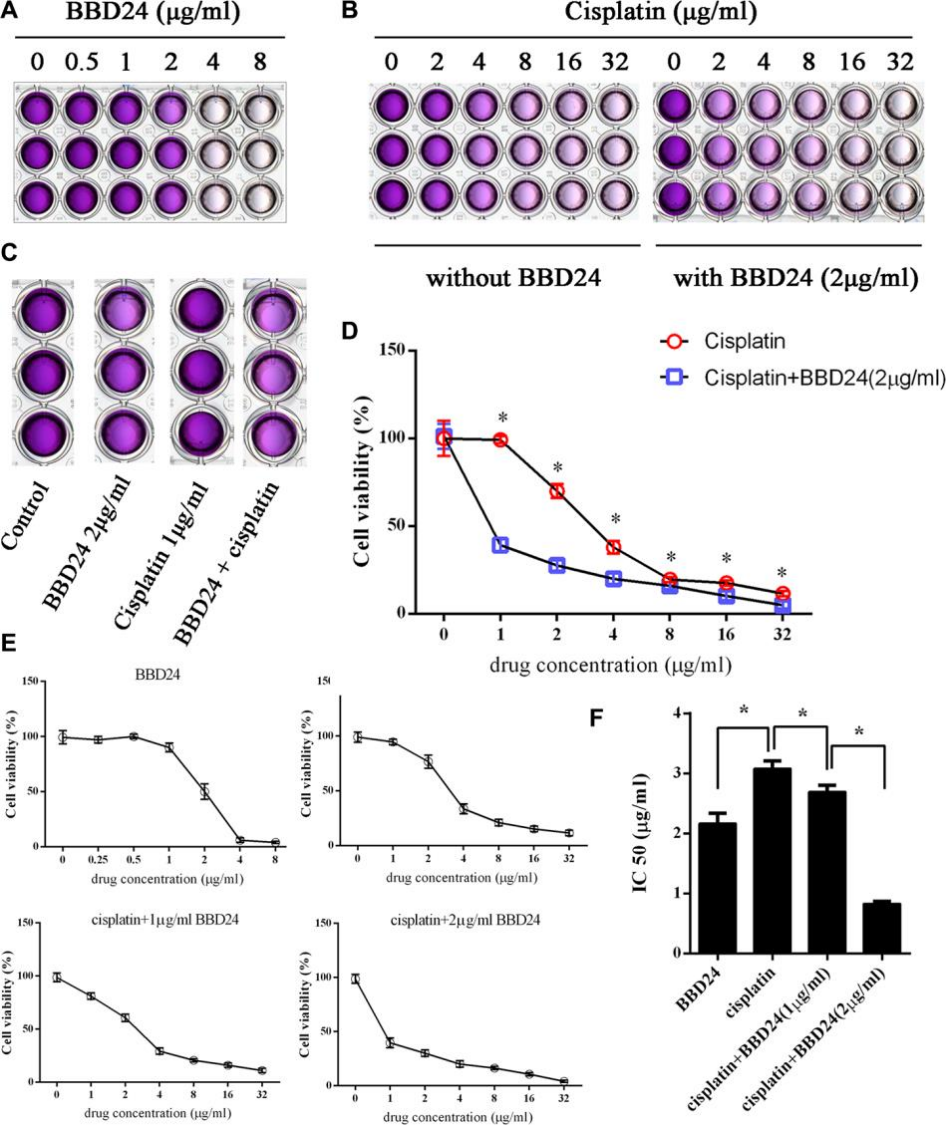
**BBD24 improved cisplatin sensitivity *in vitro*. HOS cells were treated with BBD24 and/or cisplatin for 72h, then MTT assay was used to measure the cell viability.** (**A**, **B**) HOS cells were treated with different concentrations of BBD24 or cisplatin. (**C**) HOS cells were treated with BBD24 (2 μg/ml) and/or cisplatin (1 μg/ml). (**D**) Cell viability of HOS in cisplatin group and cisplatin+BBD24 group. (**E**, **F**) IC50 of BBD24, cisplatin, cisplatin+BBD24 (1 μg/ml) and cisplatin+BBD24 (2 μg/ml). Results were expressed as means ± SD of three independent experiments. * P<0.05.

**Figure 6:**
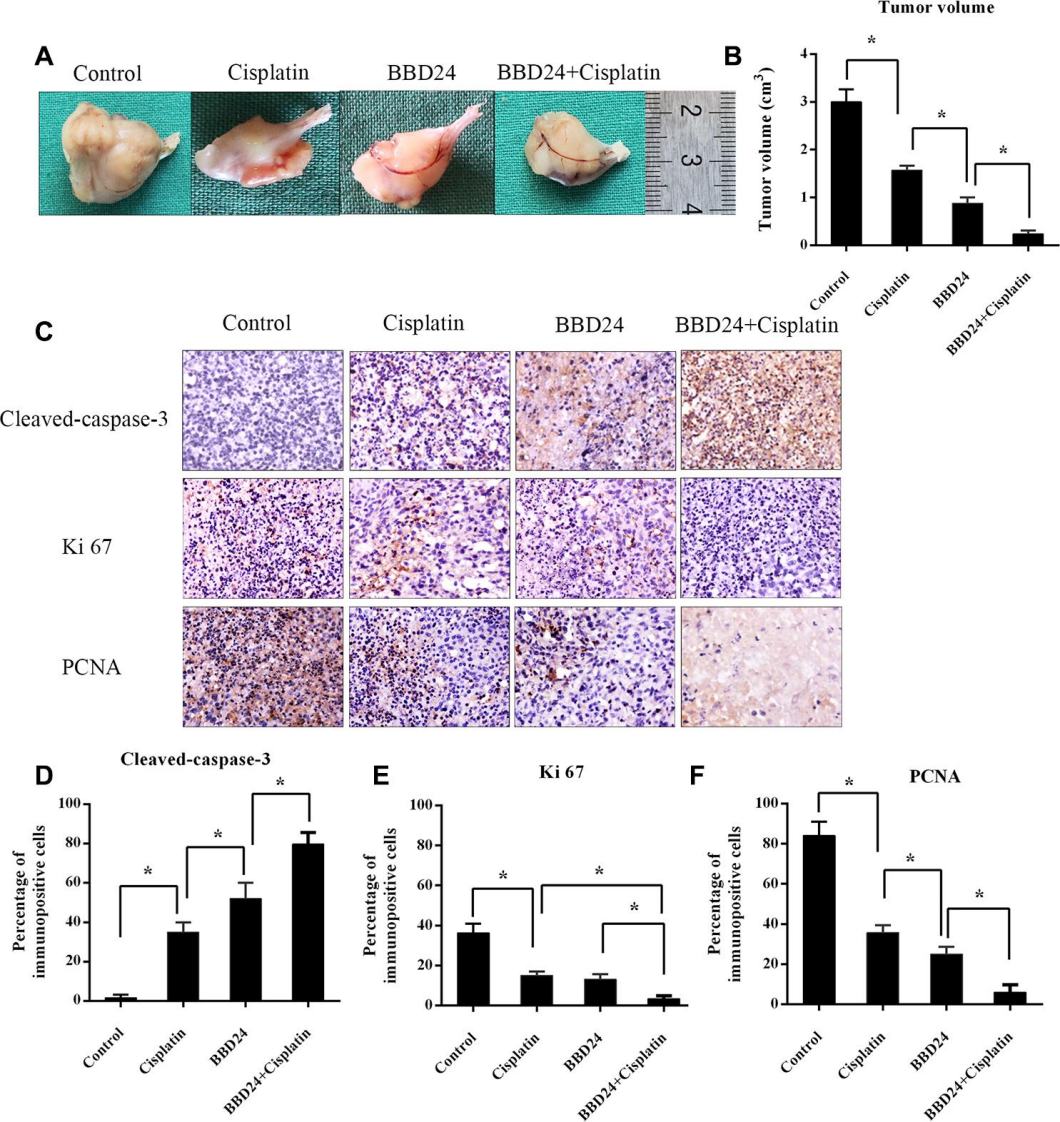
****in vivo** anti-tumor activity of BBD24. Nude mice were treated with BBD24 and/or cisplatin for 20 consecutive days.** (**A**, **B**) Tumor volumes in control, cisplatin, BBD24 and BBD24+cisplatin groups. (**C**–**F**) Immunohistochemical staining of Cleaved-caspase-3, Ki-67 and PCNA in different groups. Results were expressed as means ± SD of three independent experiments. * P<0.05.

HOS cells were treated with different concentrations of BBD24, cisplatin or both for 72 h, and the MTT assay was used to measure cell viability. We found that both BBD24 and cisplatin alone inhibited the viability of HOS cells, while combining cisplatin with 2 μg/ml BBD24 significantly increased the cisplatin sensitivity of HOS cells (Figure 5A–5D). We further applied additional concentrations of BBD24. The results showed that cisplatin combined with 2 μg/ml BBD24 achieved better results than 1 μg/ml BBD24 combined with less than half the dose of cisplatin (Figure 5E, 5F). To determine whether BBD24 could inhibit osteosarcoma *in vivo*, we treated nude mice with BBD24 and/or cisplatin for 20 consecutive days. We found that tumor volume was significantly reduced after treatment with BBD24 and/or cisplatin, while cisplatin combined with BBD24 achieved the best result (Figure 6A, 6B). Immunohistochemical staining showed similar results; both BBD24 and cisplatin induced cleaved caspase-3 expression, while the BBD24+cisplatin group had the highest rate of positive staining (Figure 6C, 6D). Ki-67 and PNCA expression was also inhibited in the BBD24 and cisplatin groups, and BBD24+cisplatin further inhibited Ki-67 and PCNA staining compared to that with BBD24 or cisplatin alone (Figure 6C, 6E, 6F). When comparing BBD24 with cisplatin, BBD24 was more effective in inhibiting tumor growth and PCNA expression (Figure 6A, 6C, 6F). BBD24 also induced more cleaved caspase-3 expression than cisplatin (Figure 6C, 6D). Taken together, our results indicated that BBD24 was able to improve the cisplatin sensitivity of OS *in vitro* and *in vivo*.

## DISCUSSION

Although a large number of studies from basic research to oncogenes have accumulated, the prognosis of, osteosarcoma is still poor. Most patients develop chemoresistance and metastasis, and approximately 80% of these patients die [31–33]. In the present study we demonstrated for the first time that BBD24, a novel derivative of the natural product berbamine, exhibited significant antitumor activity against human osteosarcoma. Our *in vitro* and *in vivo* experiments found that BBD24 was active against not only various human osteosarcoma cell lines but also primary tumor cells from patients with osteosarcoma. These data indicated that BBD24 was an effective anticancer agent against human osteosarcoma without obvious toxicity.

BBM and its derivatives were reported to have multiple targets in treating different cancers. These targets include STAT3 [34], smad3 [35], NF-κB [36], Wnt/β-catenin [26] and MEK/ERK [37]. Our previous study reported that CAMKIIγ was a target of BBM in CML [38]. How does BBD24 exert an anticancer effect on OS? In our study, we further found that BBD24 influenced multiple cancer-associated signaling networks, such as NF-κB, ERK1/2 and AKT. Nuclear factor-kappa B (NF-κB) has a pivotal role in the progression and distant metastasis of cancers, including malignant bone tumors [7–9]. The development of chemotherapy resistance and evasion from apoptosis in osteosarcoma is often correlated with constitutive nuclear NF-κB activation [9]. The RelA (p65) nuclear NF-κB subunit contributes to tumor cell survival by inducing the expression of a variety of antiapoptotic genes [9]. Extracellular regulated kinases (ERKs)-1 and -2 are members of the MAPK family of protein kinases involved in the proliferation, differentiation and apoptosis of bone cells [11, 12]. Bcl-2 is an integral outer mitochondrial membrane protein that blocks apoptotic death. One review [39] reported that Bcl-2 might be a new molecule in osteosarcoma targeted therapy, and Bcl-2 was markedly downregulated in cisplatin-treated Barkor-transfected Saos-2 cells in a dose-dependent manner [40]. Caspase-3 is a member of the caspase family, and sequential activation of caspases plays a key role in the execution phase of cell apoptosis. A previous study reported that caspase-3 was the predominant caspase protein in the apoptosis process [41]. In our study, Bcl-2, Bax, and cleaved caspase-3 were considered to take part in BBD24-induced apoptosis of OS cells.

One unique activity of BBD24, which is different from known anti-osteosarcoma agents, is its ability to trigger multiple cell death pathways by disabling multiple cancer-associated signaling networks, such as NF-κB, ERK1/2 and AKT. We have demonstrated that osteosarcoma cells exhibit at least three cell death pathways, including caspase-mediated apoptosis, necrosis and autophagic death, after exposure to BBD24. The molecular mechanism(s) by which BBD24 efficiently inhibits the growth of osteosarcoma cells is not fully understood. Previous studies reported that berbamine, the lead compound of BBD24, suppressed the expression of multidrug-resistance protein (MDR1) and Bcl-2 in K562 leukemia cells [42], caused depolarization of the mitochondrial membrane and decreased the membrane potential of HepG2 tumor cells [43]. In this study, we demonstrated that BBD24 not only potently blocks cytoplasm-to-nucleus translocation of NF-κB p65 but also inhibits the activation of AKT and ERK signaling pathways, indicating that BBD24 is a potent inhibitor of multiple cancer-associated signaling pathways. Since the NF-κB, AKT and ERK signaling pathways are aberrantly activated in osteosarcoma and frequently associated with tumor relapse or drug resistance [7–9, 11, 12, 15], the BBD24-mediated inhibitory effect on multiple cancer-associated signaling networks may be a potential strategy for combating tumor relapse or drug resistance.

As previous studies reported, berbamine and its derivatives have been proven to be broad-spectrum anticancer drugs against multiple cancers, including leukemia, liver cancer and ovarian cancer [26, 28, 44]. The typical concentrations of berbamine used in a previous study varied from 16 to 20 μg/ml, which are considerably higher than the concentrations of BBD24 used in our study. In fact, BBD24 is not the only berbamine derivative reported as an anti-osteosarcoma agent. Yang and his colleagues reported another berbamine derivative (BBMD3) that inhibited cell viability and induced apoptosis of OS cells by inducing the phosphorylation of JNK [39]. This study revealed the therapeutic potential of berbamine derivatives against osteosarcoma; however, they did not provide safety data [39]. Given that current chemotherapy agents frequently exhibit inhibitory effects on hematopoiesis, investigating the safety of chemotherapeutic agents is important. In our study, BBD24 showed no obvious effect on the growth of normal blood cells under the same conditions, and the therapeutic window was greater than 3.79 μg/ml (IC50: 8.60 μg/ml OS cells/2.27 μg/ml normal cells).

There are still some limitations to this study. More studies should be performed to further explore the molecular mechanism and to further optimize and improve the pharmacological properties of berbamine by analyzing the structure-activity relationships.

In conclusion, based on these preclinical findings, its oral bioavailability, and a favorable toxicology profile, we propose that BBD24 and its analogs have intriguing potential as novel antitumor agents against human osteosarcoma.

## MATERIALS AND METHODS

### Reagents and antibodies

**Figure 7:**
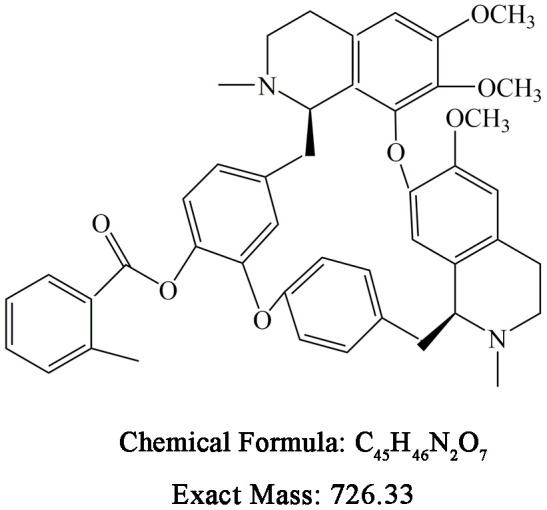
**Chemical structure and formula of BBD24.**

Berbamine (BBM) and BBD24 were obtained from Hangzhou Bensheng Pharmaceutics (Hangzhou, China), and their chemical structures and formulas are shown in Figure 7. The compound was dissolved in dimethyl sulfoxide (DMSO) at a concentration of 10 mg/ml. NF-κB p65 and histone 1 antibodies were purchased from Santa Cruz Biotechnology (Santa Cruz, CA, USA). LC3I/II and β-actin antibodies were from Sigma (St Louis, MO, USA). Caspase family, AKT, p-AKT, ERK, p-ERK, PCNA and Ki-67 antibodies were purchased from Cell Signaling Technology. The annexin V-FITC/PI apoptosis detection kit was from BD Biosciences.

### Human osteosarcoma cell lines and culture

Two human osteosarcoma cell lines, MNNG/HOS and MG63, which were provided by Cancer Institute Zhejiang University, were used in this study. OS cells were grown in DMEM supplemented with 10% FBS at 37°C in a 95% air, 5% CO_2_ humidified incubator. All cell lines were used within 20 passages.

### Primary osteosarcoma cells and normal hematopoietic cells

Institutional Review Board (IRB) approval was given by the Ethical Committee of the Second Affiliated Hospital, Zhejiang University School of Medicine (Approval number: 2017-043). Primary osteosarcoma specimens were obtained from patients who had given informed consent. Normal blood cells were also obtained from the peripheral blood of healthy volunteer donors. Mononuclear cells were isolated from the samples using Ficoll-Plaque density gradient separation. Primary osteosarcoma cells were cultured in DMEM with 10% FBS, and normal blood cells were cultured in RPMI-1640 medium with 10% FBS at 37°C in a 95% air, 5% CO2 humidified incubator. All primary cells were used within 5 passages.

### Cell viability determination

For the cell viability assay, different OS cells and blood cells were seeded at a density of 1×10^4^ cells/ml. Cells were incubated with different concentrations of BBD24 and/or cisplatin for 72 h. Cell viability was measured by MTT (3-(4,5-dimethylthiazol-2-yl)-2,5-diphenyltetrazolium bromide).

### Flow cytometry analysis

HOS cells were treated with 2 μg/ml BBD24 for 12, 24 or 48 h. Following compound treatment, the cells were harvested by trypsin, washed with PBS, and resuspended in 1× annexin-binding buffer. Cells were stained with annexin V-FITC and propidium iodide (Becton-Dickinson Company, USA). Five microliters of annexin-V-fluorescein isothiocyanate and 5 μL of propidium iodide were added to the buffer and incubated for 15 min in the dark. After that, flow cytometry analysis was performed to detect apoptotic and necrotic cells. In the flow cytometry image, the lower right quadrant represents apoptotic cells, and the upper left and upper right quadrants signify necrotic cells. We compared the apoptosis rate and necrosis rate between groups.

### Western blot analysis

HOS cells were treated with different concentrations of BBD24 for 48 h. After treatment, cellular protein was extracted using Mammalian Protein Extraction Reagent. The protein concentration was quantified using a BCA Protein Assay Kit (Thermo Scientific, USA). Nucleoprotein was used to assess the expression of nuclear p65 and histone 1. Total protein was used to assess the expression of total p65, p-ERK, ERK, p-AKT, AKT, Bcl-2, Bax, caspases, LC3I/II and β-actin. Cell homogenates were loaded into SDS-PAGE gels with concentrations ranging from 4% to 15%, separated by electrophoresis and transferred onto polyvinylidene fluoride (PVDF) membranes. The membranes were blocked with 5% fat-free milk at room temperature for 1 h and subsequently incubated overnight at 4°C with primary antibodies. Then, the membranes were washed with TBST three times and then exposed to appropriate secondary antibodies that were conjugated with IRDye 800CW at room temperature for 1 h. An Odyssey infrared imaging system (LI-COR) was used to detect the positive bands. β-Actin and histone 1 were used as cytoplasmic and nuclear loading controls, respectively.

### Migration and invasion assays

OS cells (1×10^5^) were seeded on a polycarbonate membrane insert with or without Matrigel coating (6.5 mm in diameter with 8.0 μm pores) in a Transwell apparatus (Costar, Cambridge, MA). DMEM containing 10% FBS was added to the lower chamber, while DMEM containing 0.2% BSA was added to the upper chamber. After incubation for 12 h at 37°C, the insert was washed with PBS, and cells on the top surface of the insert were removed by wiping with a cotton swab. Cells that migrated to the bottom surface of the insert were fixed with 4% paraformaldehyde and stained with 0.1% crystal violet. Cells were counted by using a microscope (Nikon Eclipse 80i; Nikon, Tokyo, Japan) and NIS-Elements D 4.50 (Nikon Instruments, Tokyo, Japan) at 200× magnification.

### Animal model and treatment

The animal model of osteosarcoma was generated as reported previously [30]. Four-week-old female nude mice (BALB/c, nu/nu; SIPPR-BK Laboratory Animal Co., Ltd., Shanghai, China) were housed under pathogen-free conditions at 26–28°C with 50–65% humidity. All animal experiments were approved by the Animal Ethics Committee of Shanghai Ninth People’s Hospital, Shanghai Jiao Tong University School of Medicine (A-2016-017). HOS cells were harvested, counted, and resuspended in PBS to a final concentration of 2 × 10^7^ cells/mL. The animals were anesthetized with 3.5% pentobarbital, and then 1 × 10^6^ HOS cells in 50 μL of PBS were injected into the proximal tibia of mice using a 25-gauge needle. After 24 hours, mice were randomly assigned to 4 groups (control group, cisplatin group, BBD24 group and BBD24+cisplatin group). BBD24 was administered orally at a dose of 5 mg/kg twice a day for 20 consecutive days. Cisplatin was intraperitoneally injected twice a week at a dose of 15 mg/kg for 20 consecutive days. The mice in the control group were given equal volumes of water. Tumor weight was measured at the end of the experiments. The tumor volumes were calculated using the formula: volume=0.2618×L×W×(L+W), where W and L represent the average width and length of the tumor, respectively [6].

### Immunohistochemistry

At the end of the experiments, the mice were sacrificed. Tumor samples were isolated from each mouse and fixed in 4% paraformaldehyde and then immunohistochemically stained for cleaved caspase-3, PCNA and Ki-67 as described previously [6].

### Statistical analysis

The results are expressed as the mean ± S.D. Differences were evaluated by one-way analysis of variance (ANOVA), followed by Duncan’s post hoc test using SPSS 21.0 (IBM, Inc.), and P values less than 0.05 were considered statistically significant.

## References

[1] Mirabello L, Troisi RJ, Savage SA. Osteosarcoma incidence and survival rates from 1973 to 2004: data from the surveillance, epidemiology, and end results program. Cancer. 2009; 115:1531–43. 10.1002/cncr.24121 19197972 PMC2813207

[2] Ferrari S, Briccoli A, Mercuri M, Bertoni F, Picci P, Tienghi A, Del Prever AB, Fagioli F, Comandone A, Bacci G. Postrelapse survival in osteosarcoma of the extremities: prognostic factors for long-term survival. J Clin Oncol. 2003; 21:710–15. 10.1200/JCO.2003.03.141 12586810

[3] Ferrari S, Smeland S, Mercuri M, Bertoni F, Longhi A, Ruggieri P, Alvegard TA, Picci P, Capanna R, Bernini G, Müller C, Tienghi A, Wiebe T, et al, and Italian and Scandinavian Sarcoma Groups. Neoadjuvant chemotherapy with high-dose ifosfamide, high-dose methotrexate, cisplatin, and doxorubicin for patients with localized osteosarcoma of the extremity: a joint study by the italian and scandinavian sarcoma groups. J Clin Oncol. 2005; 23:8845–52. 10.1200/JCO.2004.00.5785 16246977

[4] Steinmann P, Walters DK, Arlt MJ, Banke IJ, Ziegler U, Langsam B, Arbiser J, Muff R, Born W, Fuchs B. Antimetastatic activity of honokiol in osteosarcoma. Cancer. 2012; 118:2117–27. 10.1002/cncr.26434 21935912

[5] Duan L, Perez RE, Hansen M, Gitelis S, Maki CG. Increasing cisplatin sensitivity by schedule-dependent inhibition of AKT and Chk1. Cancer Biol Ther. 2014; 15:1600–12. 10.4161/15384047.2014.961876 25482935 PMC4623033

[6] Han XG, Du L, Qiao H, Tu B, Wang YG, Qin A, Dai KR, Fan QM, Tang TT. CXCR1 knockdown improves the sensitivity of osteosarcoma to cisplatin. Cancer Lett. 2015; 369:405–15. 10.1016/j.canlet.2015.09.002 26391645

[7] Nishimura A, Akeda K, Matsubara T, Kusuzaki K, Matsumine A, Masuda K, Gemba T, Uchida A, Sudo A. Transfection of NF-κB decoy oligodeoxynucleotide suppresses pulmonary metastasis by murine osteosarcoma. Cancer Gene Ther. 2011; 18:250–59. 10.1038/cgt.2010.75 21183950

[8] Kishida Y, Yoshikawa H, Myoui A. Parthenolide, a natural inhibitor of nuclear factor-kappaB, inhibits lung colonization of murine osteosarcoma cells. Clin Cancer Res. 2007; 13:59–67. 10.1158/1078-0432.CCR-06-1559 17200339

[9] Campbell KJ, Witty JM, Rocha S, Perkins ND. Cisplatin mimics ARF tumor suppressor regulation of RelA (p65) nuclear factor-kappaB transactivation. Cancer Res. 2006; 66:929–35. 10.1158/0008-5472.CAN-05-2234 16424027

[10] Lee DH, Thoennissen NH, Goff C, Iwanski GB, Forscher C, Doan NB, Said JW, Koeffler HP. Synergistic effect of low-dose cucurbitacin B and low-dose methotrexate for treatment of human osteosarcoma. Cancer Lett. 2011; 306:161–70. 10.1016/j.canlet.2011.03.001 21440986 PMC4925102

[11] Yin G, Fan J, Zhou W, Ding Q, Zhang J, Wu X, Tang P, Zhou H, Wan B, Yin G. ERK inhibition sensitizes CZ415-induced anti-osteosarcoma activity *in vitro* and *in vivo*. Oncotarget. 2017; 8:82027–36. 10.18632/oncotarget.18303 29137241 PMC5669867

[12] Qin J, Wang R, Zhao C, Wen J, Dong H, Wang S, Li Y, Zhao Y, Li J, Yang Y, He X, Wang D. Notch signaling regulates osteosarcoma proliferation and migration through erk phosphorylation. Tissue Cell. 2019; 59:51–61. 10.1016/j.tice.2019.07.002 31383289

[13] McCleese JK, Bear MD, Fossey SL, Mihalek RM, Foley KP, Ying W, Barsoum J, London CA. The novel HSP90 inhibitor STA-1474 exhibits biologic activity against osteosarcoma cell lines. Int J Cancer. 2009; 125:2792–801. 10.1002/ijc.24660 19544563

[14] Kolb EA, Kamara D, Zhang W, Lin J, Hingorani P, Baker L, Houghton P, Gorlick R. R1507, a fully human monoclonal antibody targeting IGF-1R, is effective alone and in combination with rapamycin in inhibiting growth of osteosarcoma xenografts. Pediatr Blood Cancer. 2010; 55:67–75. 10.1002/pbc.22479 20486173

[15] Zhang G, Li M, Zhu X, Bai Y, Yang C. Knockdown of Akt sensitizes osteosarcoma cells to apoptosis induced by cisplatin treatment. Int J Mol Sci. 2011; 12:2994–3005. 10.3390/ijms12052994 21686164 PMC3116170

[16] D’Incalci M, Steward WP, Gescher AJ. Use of cancer chemopreventive phytochemicals as antineoplastic agents. Lancet Oncol. 2005; 6:899–904. 10.1016/S1470-2045(05)70425-3 16257798

[17] Zhang XW, Yan XJ, Zhou ZR, Yang FF, Wu ZY, Sun HB, Liang WX, Song AX, Lallemand-Breitenbach V, Jeanne M, Zhang QY, Yang HY, Huang QH, et al. Arsenic trioxide controls the fate of the PML-RARalpha oncoprotein by directly binding PML. Science. 2010; 328:240–43. 10.1126/science.1183424 20378816

[18] Chen Y, Hu Y, Michaels S, Segal D, Brown D, Li S. Inhibitory effects of omacetaxine on leukemic stem cells and BCR-ABL-induced chronic myeloid leukemia and acute lymphoblastic leukemia in mice. Leukemia. 2009; 23:1446–54. 10.1038/leu.2009.52 19322212 PMC2726272

[19] Titov DV, Gilman B, He QL, Bhat S, Low WK, Dang Y, Smeaton M, Demain AL, Miller PS, Kugel JF, Goodrich JA, Liu JO. XPB, a subunit of TFIIH, is a target of the natural product triptolide. Nat Chem Biol. 2011; 7:182–88. 10.1038/nchembio.522 21278739 PMC3622543

[20] Küpeli E, Koşar M, Yeşilada E, Hüsnü K, Başer C. A comparative study on the anti-inflammatory, antinociceptive and antipyretic effects of isoquinoline alkaloids from the roots of turkish berberis species. Life Sci. 2002; 72:645–57. 10.1016/s0024-3205(02)02200-2 12467905

[21] Tsai TH. Analytical approaches for traditional chinese medicines exhibiting antineoplastic activity. J Chromatogr B Biomed Sci Appl. 2001; 764:27–48. 10.1016/s0378-4347(01)00277-8 11817032

[22] Xu L, Zhao XY, Wu D. [Detection of P-170 and treatment with berbamine in primary acute leukemia]. Zhejiang Da Xue Xue Bao Yi Xue Ban. 2003; 32:67–68. 12640715 10.3785/j.issn.1008-9292.2003.01.017

[23] Ren Y, Lu L, Guo TB, Qiu J, Yang Y, Liu A, Zhang JZ. Novel immunomodulatory properties of berbamine through selective down-regulation of STAT4 and action of IFN-gamma in experimental autoimmune encephalomyelitis. J Immunol. 2008; 181:1491–98. 10.4049/jimmunol.181.2.1491 18606704

[24] Jia XJ, Li X, Wang F, Liu HQ, Zhang DJ, Chen Y. Berbamine exerts anti-inflammatory effects via inhibition of NF-κB and MAPK signaling pathways. Cell Physiol Biochem. 2017; 41:2307–18. 10.1159/000475650 28456802

[25] Yang J, Zhang W. New molecular insights into osteosarcoma targeted therapy. Curr Opin Oncol. 2013; 25:398–406. 10.1097/CCO.0b013e3283622c1b 23666471

[26] Zhang H, Jiao Y, Shi C, Song X, Chang Y, Ren Y, Shi X. Berbamine suppresses cell proliferation and promotes apoptosis in ovarian cancer partially via the inhibition of Wnt/β-catenin signaling. Acta Biochim Biophys Sin (Shanghai). 2018; 50:532–39. 10.1093/abbs/gmy036 29701777

[27] Wang GY, Zhang JW, Lü QH, Xu RZ, Dong QH. Berbamine induces apoptosis in human hepatoma cell line SMMC7721 by loss in mitochondrial transmembrane potential and caspase activation. J Zhejiang Univ Sci B. 2007; 8:248–55. 10.1631/jzus.2007.B0248 17444599 PMC1838830

[28] Xu R, Dong Q, Yu Y, Zhao X, Gan X, Wu D, Lu Q, Xu X, Yu XF. Berbamine: a novel inhibitor of bcr/abl fusion gene with potent anti-leukemia activity. Leuk Res. 2006; 30:17–23. 10.1016/j.leukres.2005.05.023 16023722

[29] Xie J, Ma T, Gu Y, Zhang X, Qiu X, Zhang L, Xu R, Yu Y. Berbamine derivatives: a novel class of compounds for anti-leukemia activity. Eur J Med Chem. 2009; 44:3293–98. 10.1016/j.ejmech.2009.02.018 19285759

[30] Du L, Han XG, Tu B, Wang MQ, Qiao H, Zhang SH, Fan QM, Tang TT. CXCR1/Akt signaling activation induced by mesenchymal stem cell-derived IL-8 promotes osteosarcoma cell anoikis resistance and pulmonary metastasis. Cell Death Dis. 2018; 9:714. 10.1038/s41419-018-0745-0 29915309 PMC6006172

[31] Galluzzi L, Senovilla L, Vitale I, Michels J, Martins I, Kepp O, Castedo M, Kroemer G. Molecular mechanisms of cisplatin resistance. Oncogene. 2012; 31:1869–83. 10.1038/onc.2011.384 21892204

[32] Zhang W, Li Q, Song C, Lao L. Knockdown of autophagy-related protein 6, beclin-1, decreases cell growth, invasion, and metastasis and has a positive effect on chemotherapy-induced cytotoxicity in osteosarcoma cells. Tumour Biol. 2015; 36:2531–39. 10.1007/s13277-014-2868-y 25427639

[33] Gorlick R, Khanna C. Osteosarcoma. J Bone Miner Res. 2010; 25:683–91. 10.1002/jbmr.77 20205169

[34] Hu B, Cai H, Yang S, Tu J, Huang X, Chen G. Berbamine enhances the efficacy of gefitinib by suppressing STAT3 signaling in pancreatic cancer cells. Onco Targets Ther. 2019; 12:11437–51. 10.2147/OTT.S223242 31920333 PMC6935307

[35] Liang Y, Qiu X, Xu RZ, Zhao XY. Berbamine inhibits proliferation and induces apoptosis of KU812 cells by increasing Smad3 activity. J Zhejiang Univ Sci B. 2011; 12:568–74. 10.1631/jzus.B1000230 21726064 PMC3134845

[36] Liang Y, He X, Li X, Zhang X, Zhang X, Zhang L, Qiu X, Zhao X, Xu R. 4-chlorbenzoyl berbamine, a novel derivative of the natural product berbamine, potently inhibits the growth of human myeloma cells by modulating the NF-κB and JNK signalling pathways. Cancer Invest. 2016; 34:496–505. 10.1080/07357907.2016.1235709 27768381

[37] Mou L, Liang B, Liu G, Jiang J, Liu J, Zhou B, Huang J, Zang N, Liao Y, Ye L, Liang H. Berbamine exerts anticancer effects on human colon cancer cells via induction of autophagy and apoptosis, inhibition of cell migration and MEK/ERK signalling pathway. J BUON. 2019; 24:1870–75. 31786849

[38] Gu Y, Chen T, Meng Z, Gan Y, Xu X, Lou G, Li H, Gan X, Zhou H, Tang J, Xu G, Huang L, Zhang X, et al. CaMKII γ, a critical regulator of CML stem/progenitor cells, is a target of the natural product berbamine. Blood. 2012; 120:4829–39. 10.1182/blood-2012-06-434894 23074277 PMC4507036

[39] Yang F, Nam S, Zhao R, Tian Y, Liu L, Horne DA, Jove R. A novel synthetic derivative of the natural product berbamine inhibits cell viability and induces apoptosis of human osteosarcoma cells, associated with activation of JNK/AP-1 signaling. Cancer Biol Ther. 2013; 14:1024–31. 10.4161/cbt.26045 24025361 PMC3925657

[40] Zhao Z, Tao L, Shen C, Liu B, Yang Z, Tao H. Silencing of barkor/ATG14 sensitizes osteosarcoma cells to cisplatin-induced apoptosis. Int J Mol Med. 2014; 33:271–76. 10.3892/ijmm.2013.1578 24337183 PMC3896476

[41] Wang L, Jin F, Qin A, Hao Y, Dong Y, Ge S, Dai K. Targeting Notch1 signaling pathway positively affects the sensitivity of osteosarcoma to cisplatin by regulating the expression and/or activity of caspase family. Mol Cancer. 2014; 13:139. 10.1186/1476-4598-13-139 24894297 PMC4110525

[42] Wei YL, Xu L, Liang Y, Xu XH, Zhao XY. Berbamine exhibits potent antitumor effects on imatinib-resistant CML cells *in vitro* and *in vivo*. Acta Pharmacol Sin. 2009; 30:451–57. 10.1038/aps.2009.19 19270722 PMC4002272

[43] Wang GY, Lv QH, Dong Q, Xu RZ, Dong QH. Berbamine induces fas-mediated apoptosis in human hepatocellular carcinoma HepG2 cells and inhibits its tumor growth in nude mice. J Asian Nat Prod Res. 2009; 11:219–28. 10.1080/10286020802675076 19408145

[44] Meng Z, Li T, Ma X, Wang X, Van Ness C, Gan Y, Zhou H, Tang J, Lou G, Wang Y, Wu J, Yen Y, Xu R, Huang W. Berbamine inhibits the growth of liver cancer cells and cancer-initiating cells by targeting ca²^+^ /calmodulin-dependent protein kinase II. Mol Cancer Ther. 2013; 12:2067–77. 10.1158/1535-7163.MCT-13-0314 23960096 PMC3808882

